# Prevalence of Trachomatous Trichiasis in Ten Evaluation Units of Embu and Kitui Counties, Kenya

**DOI:** 10.1080/09286586.2021.1986549

**Published:** 2022-01-17

**Authors:** D Ilako, E Barasa, M Gichangi, S Mwatha, T Watitu, J Bore, A Rajamani, R Butcher, RM Flueckiger, A Bakhtiari, R Willis, AW Solomon, EM Harding-Esch, SH Matendechero

**Affiliations:** a Department of Ophthalmology, University of Nairobi, Nairobi, Kenya; b Ophthalmic Services Unit, Ministry of Health, Nairobi, Kenya; c Neglected Tropical Diseases Unit, Ministry of Health, Nairobi, Kenya; d Kenya National Bureau of Statistics, Ministry of Planning, Nairobi, Kenya; e Clinical Research Department, London School of Hygiene & Tropical Medicine, London, UK; f RTI International, Atlanta, GA, USA; g Task Force for Global Health, Atlanta, GA, USA; h Department of Control of Neglected Tropical Diseases, World Health Organization, Geneva, Switzerland

**Keywords:** Elimination, Kenya, prevalence, trachoma, trachomatous trichiasis

## Abstract

**Background:**

Late-stage blinding sequalae of trachoma such as trachomatous trichiasis (TT) typically take decades to develop and often do so in the absence of ongoing ocular *Chlamydia trachomatis* infection. This suggests that most TT risk accumulates in early life; as a result, population-level TT incidence and prevalence can remain high years after *C. trachomatis* transmission among children has decreased. In Embu and Kitui counties, Kenya, the prevalence of trachomatous inflammation – follicular is low in children. In this survey, we set out to determine the prevalence of TT in ten evaluation units (EUs) in these counties.

**Methods:**

We undertook ten cross-sectional prevalence surveys for TT. In each EU, people aged ≥15 years were selected by a two-stage cluster sampling method and examined for TT. Those with TT were asked questions on whether they had been offered management for it. Prevalence was adjusted to the underlying age and gender structure of the population.

**Results:**

A total of 18,987 people aged ≥15 years were examined. Per EU, the median number of examined participants was 1,656 (range: 1,451 − 3,016) and median response rate was 86% (range: 81 − 95%). The prevalence of TT unknown to the health system in people aged ≥15 years was above the threshold for elimination (≥0.2%) in all ten EUs studied (range: 0.2–0.7%). TT was significantly more common in older than younger individuals and in women than in men.

**Discussion:**

Provision of surgical services should be strengthened in Embu and Kitui counties of Kenya to achieve the World Health Organization threshold for eliminating TT as a public health problem.

## Introduction

Trachoma is the leading infectious cause of blindness worldwide and is endemic in >40 countries.^1,2^ Ocular infection with *Chlamydia trachomatis* leads to conjunctival inflammation. Recurrent rounds of inflammation over time result in scarring of the upper sub-tarsal conjunctiva which can cause the eyelid to turn inward, resulting in eyelashes rubbing against the eyeball. This painful state is called trachomatous trichiasis (TT) and can damage the cornea, leading to impairment of vision or blindness.^3^ Because of the number of rounds of infection and inflammation needed for clinically relevant scarring to develop, TT is much more common in older people.^4,5^

Trachoma has been targetted for elimination as a public health problem,^6,7^ which is achieved through a package of interventions known as the SAFE strategy (surgery, antibiotics, facial cleanliness and environmental improvement).^8^ To determine where these interventions need to be implemented, population-based surveys are required to establish the prevalence of trachoma in a given evaluaution unit (EU) or district (defined as an adminstrative division with a population between 100,000–250,000^9^). One of three key targets for trachoma to be validated as having been eliminated as a public health problem by the World Health Organization (WHO) is that the prevalence of TT unknown to the health system (i.e. individuals with TT who have not previously been offered management^9^) must be <0.2% in those aged ≥15 years in all formerly-endemic EUs.^7^

Trachoma was first confirmed as an issue of public health concern in Kenya in 2004 when the first baseline survey was carried out.^10^ Subsequent baseline surveys followed in 2007–2012. Of the 47 counties in Kenya, 12 counties, with a combined population of approximately 7 million people, were confirmed to be trachoma-endemic: Turkana, Kajiado, Samburu, Laikipia, Marsabit, Isiolo, Kitui, Embu, Meru, Narok, West Pokot and Baringo. In 2013, the Ministry of Health estimated there to be 41,500 individuals in these 12 counties who had TT and were at risk of progressive blindness, requiring surgery to prevent it. The Kenya Trachoma Elimination Programme (KTEP) was therefore established. Initiated in 2014, the 5-year programme was given the ambitious goal of eliminating trachoma as a public health problem by 2019, using the SAFE strategy.

TT is the sight-threatening stage of trachoma, so timely and appropriate management of individuals with TT is a priority of trachoma elimination programmes. Between its inception and December 2019, KTEP managed 32,598 people with TT. This included 2,094 and 178 surgeries conducted in Kitui and Embu counties, respectively, To allow for proper planning of future work, it became imperative to know the current status of TT prevalence at county level.

Typically, surveys to estimate trachoma prevalence enroll people aged ≥1 year and assess both TF prevalence in 1–9-year-olds and TT prevalence in ≥15-year olds at the same time. However, in certain contexts, surveys that include examination of both children and adults are unnecessary, for example, where the TF prevalence in 1–9-year-olds is known to be <5% but TT may still constitute a public health problem.^11^ Since most conjunctival *C. trachomatis* infection occurs in children, individuals probably accumulate most of the risk of developing TT in early life but do not manifest it until many years later: the presence of low TF prevalence but moderate-to-high TT prevalence may reflect recent decreases in *C. trachomatis* transmission intensity and does not constitute an epidemiological paradox like the high-TF, low-TT situation seen in Melanesia.^12^

In 2012, the baseline prevalence of TF in 1–9-year-olds was <5% (range: 0.0–4.8%) in all 9 surveyed EUs of Kitui and Embu counties, whereas the prevalence of TT was ≥0.2% in those aged ≥15 years in 7/9 EUs (Table 1). Although Mwingi West and Mwingi Central each had TT prevalences of <0.2% at baseline, it was noted post-survey that there was manifest demand for TT surgical services in local residents. The aim of the current round of surveys was, therefore, to assess TT prevalence in those aged ≥15 years in EUs across these two counties to determine proximity to the WHO-recommended elimination threshold for TT.

**Table 1.** Summary of survey demographics of ten evaluation units surveyed for trachomatous trichiasis in Kenya (July 2018–April 2019).

County	Constituency	2012 prevalence estimate^†^		EU ID	Number of clusters selected	Number of house-holds enumerated	Number of people aged ≥15 years enumerated	Number of people aged ≥15 years absent	Number of people aged ≥15 years who refused	Number of people aged ≥15 years examined (response rate, %)	Number of people aged ≥15 years examined who were female (%)
		TF in 1–9 years (%)	TT in ≥15 years (%)								
Embu	Mbeere North	0.0	0.6	50133	30	1049	3189	162	10	3016 (95)	1713 (57)
	Mbeere South	0.2	0.3	50058	30	900	1980	291	7	1682 (85)	975 (58)
Kitui	Kitui Central	0.4	0.7	50059	30	899	2043	239	7	1797 (88)	1098 (61)
	Kitui East	0.0	0.4	50134	30	1046	2993	202	2	2789 (93)	1630 (58)
	Kitui South	1.2	1.8	50061	30	901	2088	255	9	1824 (87)	1104 (61)
	Kitui Rural	*	*	50062	30	900	1902	267	6	1629 (86)	984 (60)
	Kitui West	0.8	1.5	50063	30	902	1920	306	10	1603 (83)	965 (60)
	Mwingi Central	0.5	0.1	50064	30	899	1950	346	7	1597 (82)	959 (60)
	Mwingi North	4.8	0.8	50065	30	897	1770	313	6	1451 (82)	856 (59)
	Mwingi West	0.5	0.1	50066	30	900	1985	380	6	1599 (81)	988 (62)
* Kitui Rural was part of Kitui West when the 2012 surveys were conducted. ^†^ Unpublished data, Kenya Trachoma Elimination Programme. EU: evaluation unit. TF: trachomatous inflammation─follicular; TT: trachomatous trichiasis.											

## Methods

Study ethics and participant consent

The surveys were approved by the Kenya Medical Research Institute. Ethical approval for Tropical Data to support these surveys was provided by the London School of Hygiene & Tropical Medicine Ethics Committee (reference: 16105). Consent forms were translated into Kamba, Embu and Swahili and verbally explained to all participants in their local language. Written informed consent to take part in the survey was provided by those aged ≥18 years, or by a parent or guardian for those aged 15–17 years. Illiterate individuals were permitted to provide verbal consent to take part. Consent for examination was also recorded in the Tropical Data app used for data collection. Individuals identified as having TT or other significant ocular pathology (e.g. cataract) were referred to local ophthalmic services for appropriate management.

Study design and participant selection

This study followed a validated methodology for population-based prevalence surveys for TT, referred to as a “TT-only” survey.^11^ For the purposes of this survey, each EU was planned to cover an administrative unit of 100,000 − 250,000, which was equivalent to a constituency in Kenya.^9^ The EUs selected to be surveyed were, for the most part, EUs in which 2012 baseline surveys had found the TF prevalence to be <5% but the TT prevalence to be ≥0.2%. There were two exceptions (Mwingi West and Mwingi Central) for which the 2012 TF prevalence was <5% and the 2012 TT prevalence was <0.2% but where subsequent outreach services had continued to identify TT cases, as noted above; TT-only surveys were also planned here.

We determined that a sampling frame incorporating 3,382 individuals aged ≥15 years would be required in each EU to estimate, with 95% confidence, a TT prevalence in those aged ≥15 years of 0.2 ± 0.2%. That target accounted for disease clustering by incorporating a design effect of 1.47 and for non-response by incoporating an inflation factor of 1.2; the target sample size of ≥15-year-olds was 2,818.^11^

Individuals were selected using a two-stage cluster selection approach. The primary sampling unit, referred to hereafter as the cluster, was the village; the secondary sampling unit was the household. In the first stage, clusters were systematically selected from each EU with selection probability proportional to village population size. In the second stage, households were selected using compact segment sampling. For these surveys, a household was defined as a group living together and sharing meals. Consenting individuals aged ≥15 years in selected households were eligible for inclusion.

The number of households per cluster was pre-determined as 30, the number a team could routinely visit in one day. The number of clusters was determined as the smallest number needed to yield the sample size, assuming 30 households would be surveyed per cluster. Based on the most recent census data, a mean of 3.8 individuals aged ≥15 years were expected to be resident per household; therefore, 30 clusters needed to be surveyed to achieve the target sample size; this is the maximum number of clusters recommended by WHO for TT-only surveys.^11^ To maximise enrolment, field teams notified clusters of their visit in advance of their arrival and also made one return visit to households where one or more adults were absent at the first visit.

Clinical examinations

Grader trainees were trained to identify the presence of TT according to the simplified trachoma grading system^13^ and undertook an objective structured clinical examination (OSCE) for TT grading under the assessment of an experienced Tropical Data-certified grader trainer. The OSCE involved stations on practical examination methods (loupe use, hand cleanliness, patient placement, upper and lower eyelid margin examination), grading of TT photo sets and eversion of upper eyelids to examine the tarsal conjunctivae for trachomatous scarring (TS).^13^ To ensure grader quality, graders needed to complete all training components and pass the OSCE to be certified to participate in the field survey.^14^ Ten field teams were supported by two supervisory teams.

All consenting survey participants were examined for evidence of TT using a 2.5× magnifying loupe. The eyelid of origin of trichiatic eyelashes was recorded. Those identified to have TT were subsequently examined for TS through eversion of the upper eyelid. Where an individual had TT but the eyelid could not be everted, they were considered to have TS. Individuals found to have TT were asked if they had previously been offered surgery or epilation.

Definitions

A recommendation to change the definition of TT was made at the 4th Global Scientific Meeting on Trachoma in November 2018.^15,16^ The survey series presented here was planned and commenced before that meeting. Therefore, definitions in this manuscript are as follows: first, TT refers to any eyelash touching the eyeball or evidence of removal of inturned eyelashes, regardless of whether that eyelash originates from the upper or lower eyelid, as described in the WHO simplified grading system as published in 1987.^13^ Where eyelashes from the upper eyelid touched the eyeball, this has been specified in the results. Individuals with TT who had not been offered surgery, or who could not remember whether they had been offered surgery, were classified as “unknown to the health system”, in accordance with WHO guidance.^11,17^ The presence or absence of TS is disregarded in the general definition of TT in this manuscript.

Data recording, storage and analysis

Field data were collected into Android devices using the Secure Data Kit-based Tropical Data app. Data were encrypted and uploaded through a secure connection to the Tropical Data server (www.tropicaldata.org). The proportion of TT-affected individuals in each five-year age band up to the age of 79 years, and for those aged ≥80 years, was determined at the cluster level. (Five-year age bands were unavailable in census data for the population aged ≥80 years.) To account for non-random differences in participant availability, these estimates were weighted at cluster level according to population composition, in the same age and gender groups, from the most recent (2009) public census data.^18^ The EU-level prevalence was then determined as the mean of the age- and gender-adjusted cluster-level prevalences. The 95% confidence interval around each EU-level prevalence estimate was calculated by resampling, with replacement, the cluster-level adjusted prevalence estimates 10,000 times.^19^ Association between TT, age and gender was assessed using a mixed-effects multivariable binomial logistic regression model (lme4::glmer function in R). EU, cluster and household were tested as random-effect variables. The relative contribution of each variable was assessed by comparing models with and without each variable using likelihood ratio tests.

## Results

Study population and recruitment

Fieldwork took place between July 2018 and April 2019. Of the 21,820 people aged ≥15 years enumerated in selected households across all 10 EUs, 18,987 were examined. Of those who were enumerated but not examined, the main reason for non-participation (reported for 97% of non-respondents) was absence at the time of survey teams’ visits. The median number of participants examined per EU was 1,656 (range: 1,451 − 3,016). The median response rate per EU was 86% (range: 81 − 95%). Table 1 contains an EU-level summary of the surveyed populations.

Clinical findings

**Figure 1.**
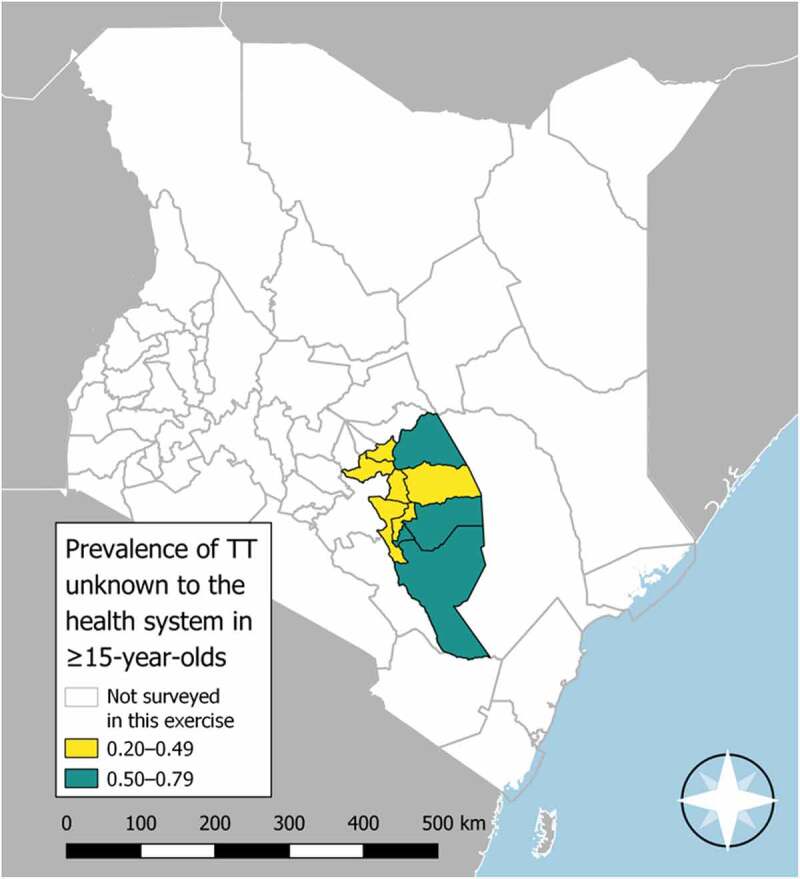
Trachomatous trichiasis (TT) prevalence unknown to the health system in people aged ≥15 years in ten evaluation units of Kenya (July 2018–April 2019). The boundaries and names shown and the designations used on this map do not imply the expression of any opinion whatsoever on the part of the World Health Organization concerning the legal status of any country, territory, city or area or of its authorities, or concerning the delimitation of its frontiers or boundaries.

A total of 262 TT cases were identified across all EUs. 198 (76%) of those cases were bilateral. 245 (94%) had at least one trichiatic eyelash coming from the upper eyelid. 256 (98%) of TT cases reported not having been offered surgery for their TT. Table 2 shows the age- and gender-adjusted prevalence of TT unknown to the health system in each EU. Also presented are the number of TT cases affecting the upper eyelid, the number of TT cases with TS and the number who had previously reported being operated on. The prevalence of TT unknown to the health system in those aged ≥15 years is displayed in Figure 1.

**Table 2.** Trachomatous trichiasis prevalence in ten evaluation units surveyed in Kenya (July 2018–April 2019).

County	Constituency	Number of people aged ≥15 years examined	Number of people aged ≥15 years with TT	Number of people aged ≥15 years with TT (upper eyelid only)	Number of people aged ≥15 years with TT with TS	Number of people aged ≥15 years with post-operative TT	Number of people aged ≥15 years with TT unknown to the health system	Age- and gender-adjusted prevalence of TT unknown to the health system in those aged ≥15 years (95% CI)
Embu	Mbeere North	3016	27	27	18	1	25	0.5 (0.2–0.6)
	Mbeere South	1682	16	14	15	0	16	0.3 (0.2–0.4)
Kitui	Kitui Central	1797	23	20	20	0	22	0.3 (0.2–0.4)
	Kitui East	2789	33	30	24	0	33	0.5 (0.2–0.8)
	Kitui South	1824	25	23	18	0	25	0.5 (0.3–0.7)
	Kitui Rural	1629	34	31	29	0	34	0.5 (0.3–0.8)
	Kitui West	1603	17	15	13	0	17	0.3 (0.2–0.5)
	Mwingi Central	1597	27	27	24	0	27	0.4 (0.2–0.7)
	Mwingi North	1451	45	43	41	1	42	0.7 (0.5–0.9)
	Mwingi West	1599	15	15	14	0	15	0.2 (0.1–0.4)
CI: confidence interval; TS: trachomatous scarring; TT: trachomatous trichiasis (according to simplified grading scheme, one or more eyelash touching the eyeball or evidence of recent removal of in-turned eyelashes.^13^)								

Association between trachomatous trichiasis, age and gender

**Figure 2.**
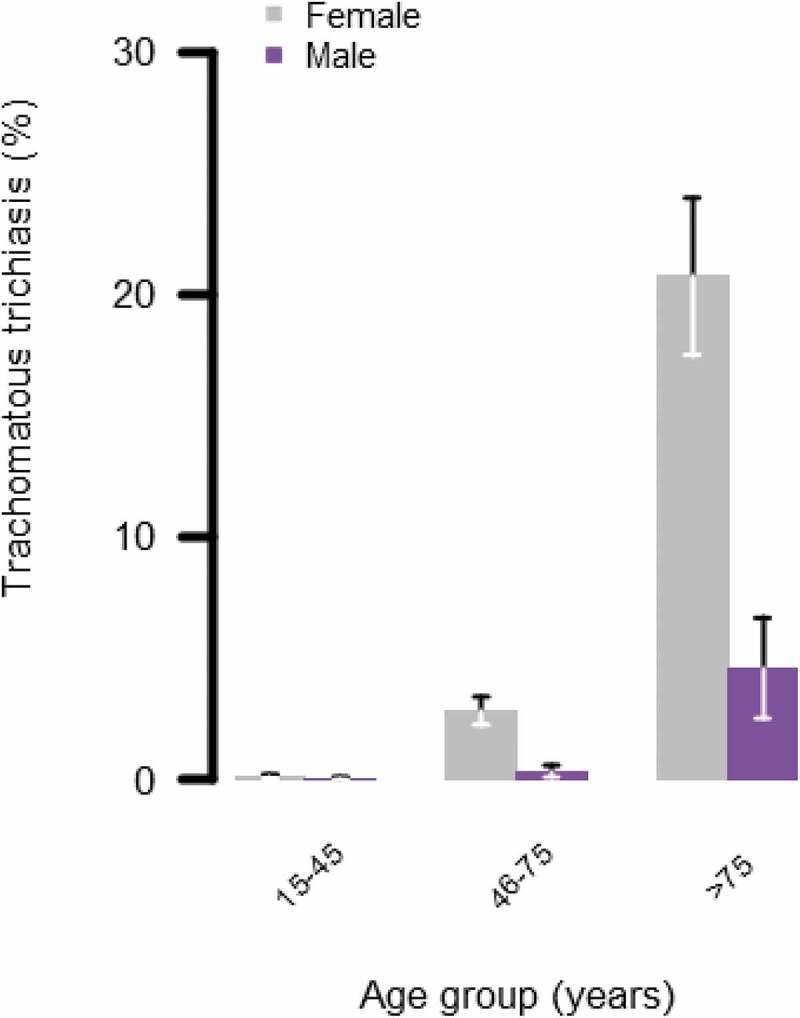
Percentage of men and women with trachomatous trichiasis (management status not specified) separated by age group. Whiskers represent 95% confidence intervals around age group-specific percentage estimates. Data collected during population-based prevalance surveys of ten evalution units in Kitui and Embu counties, Kenya (July 2018–April 2019).

Optimal mixed-effect model fit was achieved with EU and cluster as random-effect variables. TT was signficantly more common in females than in males (adjusted odds ratio [aOR]: 6.1, 95% confidence interval [CI]: 4.1–9.1, *p* < .001) and significantly more common in those aged 46 − 75 years and those aged >75 years than those aged 15 − 45 years (reference level: 15 − 45 years; aOR for 46 − 75 years: 18.9 [95% CI: 10.6 − 33.8]; aOR for those aged >75 years: 194.2 [95% CI: 108.6 − 347.3]). The majority of TT cases were found in people aged >75 years (172/245, 70%). The distribution of TT cases among males and females of different ages is shown in Figure 2.

## Discussion

The survey data presented here suggest the prevalence of TT unknown to the health system was above the threshold for elimination as a public health problem (<0.2% in people aged ≥15 years^7^) in all ten surveyed EUs of Embu and Kitui counties. This suggests that an ongoing public health response to TT is warranted. The response could include, for example, active case finding and management, and improvement of awareness of surgical services. Planning such services can be supported by rough estimation of case numbers. If we take the combined estimated population of each of these EUs in 2018 and assume 58% of that population is ≥15 years (as indicated by 2009 census data^18^) we estimate that there are ~3,900 individuals living in these EUs with TT who have yet to be identified and offered management by the health system, although the inaccuracy in such estimates is well described.^20^ It is also notable that, of the 2,272 people offered trichiasis surgery in Embu and Kitui counties by the KTEP up to 2019, there were 64 individuals who refused. It is important to understand the reasons behind these refusals to support programme staff to maximise uptake. It should also be noted that Kitui has roughly double the population of Embu, but has carried out more than ten-times the number of trichiasis operations. Embu has one of the lowest numbers of surgeries conducted of any county. This highlights the necessity to ensure programmes are delivered equitably.

This study has some limitations. The target sample size of 2818 examined adults was not met in nine of the 10 surveyed EUs (Table 2). The lower-than-expected precision with which TT prevalence has been estimated in these EUs is corresondingly lower (Table 2) than we aimed to achieve in our method statement. However, TT-only survey guidelines are explicit in their advice to limit the number of clusters to be visited to 30 where >30 clusters are expected to be needed to acheive the sample size target. This recommendation is supported by simulation studies of over-sampled datasets, which suggest that surveying >30 clusters does not yield a useful improvement in precision^11^; in our surveys, 30 clusters were sampled in all EUs. Second, TT was graded according to WHO’s original simplified grading system. The system has recently been amended so that trichiasis is only regarded as “trachomatous” when the inturned eyelashes come from the upper eyelid.^16^ The proportion of TT cases with upper eyelid involvement in this series of surveys was 94% (Table 2), but as questions on management were not specifically disaggregated by eyelid, it is not possible to determine the prevalence of upper-eyelid TT unknown to the health system. This difference in grading should be considered when comparing these estimates to future estimates. Finally, two constituencies of Embu county (Manyatta and Runyenjes) were not surveyed in this exercise. However, these constituencies are in a different climatic zone to the other surveyed constituencies, have better access to a major water tower at Mount Kenya and do not regularly report cases of trachoma. They are, therefore, considered to be at low risk of high trachoma prevalence.

It is difficult to use these prevalence estimates to make inferences about the success of any interventions which have taken place between the 2012 and 2018 survey rounds, for a number of reasons. The limitations of the current exercise have been described in the paragraph above, but additional factors also make comparison problematic. First, assuming a constant incidence of TT and a constant mortality rate in the general population, it would be reasonable to assume that prevlance of TT would remain stable. However, in reality, both TT incidence and population mortality are likely to vary with time based on a range of factors. A systematic review evaluating the incidence of TT in trachoma-enemic communities produced estimates of between 0.06–3.0% per year, depending on location, age, gender and conjunctival scarring status,^21^ though data that inform this range come from only three countries.^22–26^ Estimates from the World Bank suggest mean life expectancy is increasing in Kenya (data.worldbank.org), which could lead to an expansion in the population of older age groups at highest risk of TT. Second, neither the 2012 nor 2018 surveys were designed to estimate prevalence with sufficiently high precision to enable comparisons if differences were small. Third, given the low number of cases, small differences in survey design (such as the method for undertaking adjustment for age and gender) between 2012 and 2018 could have a disproportionaly large effect on the estimated prevalence. Face-value comparisons between these two survey estimates are therefore unlikely to be informative. It is, however, notable that 98% of individuals with TT reported not previously having been offered management for their TT, despite the KTEP conducting >32,000 TT surgeries nationwide between 2014 and 2018.

These data highlight the need for implementation of robust systems to identify and manage incident cases of TT. Investment in such systems now would also help the programme get a head-start on the third criterion for trachoma’s elimination as a public health problem.^7^
